# A method of partially overlapping point clouds registration based on differential evolution algorithm

**DOI:** 10.1371/journal.pone.0209227

**Published:** 2018-12-21

**Authors:** Xuetao Zhang, Ben Yang, Yunhao Li, Changle Zuo, Xuewei Wang, Wanxu Zhang

**Affiliations:** 1 National Engineering Laboratory for Visual Information Processing and Applications, Xi’an, Shaanxi 710049, China; 2 School of Electronic and Information Engineering, Xi’an Jiaotong University, Xi’an, Shaanxi 710049, China; 3 College of Computer Science and Technology, Zhejiang University, Hangzhou, Zhejiang 310027, China; 4 School of Information Science and Techonology, Northwest University, Xi’an 710127, China; Huazhong University of Science and Technology, CHINA

## Abstract

3D point cloud registration is a key technology in 3D point cloud processing, such as 3D reconstruction, object detection. Trimmed Iterative Closest Point algorithm is a prevalent method for registration of two partially overlapping clouds. However, it relies heavily on the initial value and is liable to be trapped in to local optimum. In this paper, we adapt the Differential Evolution algorithm to obtain global optimal solution. By design appropriate evolutionary operations, the algorithm can make the populations distributed more widely, and keep the individuals from concentrating to a local optimum. In the experiment, the proposed algorithm is compared with existing methods which are based on global optimization algorithm such as Genetic Algorithm and particle filters. And the results have demonstrated that the proposed algorithm is more robust and can converge to a good result in fewer generations.

## Introduction

With the development of the depth-sensing technology, such as Kinect and 3D LiDAR, the acquisition of 3D point cloud becomes more convenient. Furthermore, the 3D point cloud processing attracts more and more attention from research and industry fields [1–6]. Point cloud registration is one of the key technologies in 3D point cloud processing. The goal of registration is to obtain the spatial transformation between two different point clouds, which is quite useful in 3D reconstruction and object detection.

Numbers of registration methods are based on the iterative closest point (ICP) [7, 8] algorithm as its high efficiency and accuracy. However, the classical ICP algorithm has some disadvantages which limit its usage in real world applications. Firstly, the speed and accuracy of convergence rely on the initial value heavily. Secondly, when the two point sets are partially overlapped, which means for some points in one set we cannot find corresponding points in the other set, the registration result becomes worse. And the algorithm may fail, if the overlapped portion is small.

Trimmed Iterative Closest Point (TrICP) [9, 10] algorithm has been proposed to handle the partially overlapping point sets registration problem. It estimates the rotation and translation between two sets as well ase the overlapping rate. Therefore, in each iteration it finds the correspondences only between overlapped points, and then updates the transformation using the corresponding point pairs. Accurately, when the overlapping rate nears one, the TrICP algorithm approaches the ICP algorithm. However, TrICP still need an appropriate initial value for iterative optimization procedure. Otherwise the algorithm would possibly converge to a local optimum. Moreover, TrICP is time consuming, because it needs to visit all possible overlap rate and search the one which get the best registration result.

The Differential Evolution (DE) [11] algorithm was proposed in 1997 by Rainer Storn and Kenneth Price on the basis of evolutionary ideas such as genetic algorithms. Substantially, it is a multi-objective optimization algorithm for obtaining the optimal solution in a multidimensional space. The source of differential evolution is the early proposed genetic algorithm (Genetic Algorithm, GA) [12], which simulates crossover, mutation, and reproduction in genetics to design genetic operators. Comparing with GA, the DE algorithm firstly generates the intermediate population based on the difference among the current population, and then obtain the new generation by competing between the intermediate population and the parent population. The DE algorithm has many advantages, such as high converging speed, fewer parameters.

To overcome disadvantages of traditional TrICP algorithm, the current paper proposed a global optimization method for TrICP based on DE algorithm. Through the appropriate design of the evolutionary operation, the population can be distributed widely, and increasing the probability getting the global optimal solution. It is efficient and accurate, even when the overlapping rate is low.

## Related works

The original ICP algorithm relies heavily on the initial estimation of the transformation between two point clouds. And it also requires the two clouds should be close to each other. Moreover, the algorithm may fail when there the noise is significant or the number of outlier is large [13]. In the literature, numerous efforts have been made to tackle these problems.

Tsin and Kanade [14] proposed a Kernel Correlation algorithm which represented the point clouds as probability distribution, and the distance between the clouds was measured by the similarity of the distribution. However, the computation cost is very high as each point in one cloud should be compared with all points in the other.

The filter-based approaches have also been proposed to solve the local optimal solution problem. Unscented Particle Filter (UPF) is able to register small point clouds [15]. This method requires great number of particles to obtain accurate registration result. Thus, when the amount of points is large, the computational cost will be high. Although the Unscented Kalman Filter (UKF) could overcome this shortcoming, but it is constrained by unimodal distribution assumption of the state vector. Therefore, the algorithm would fail when the distribution is complex. Zhu, et al. proposed to use particle filter for rigid registration [16]. However, this algorithm is time consuming because it needs lots of particles to obtain accurate result.

TrICP introduces an overlapping rate into a least square function in order to improve the robustness to partial overlapping. However, the computation is slow. Phillips et al. [17] presented Fractional ICP (FICP) algorithm to speed up the process of registration. The algorithm computes the best correspondence and overlapping rate simultaneously. But FICP relies heavily on hyper-parameters which may lead registration to failure. Gold [18] proposed the Robust Point Matching (RPM) method, which applies the annealing algorithm to reduce the exhaustive search time. However, when noise is significant or some structural missing exist, RPM would fail.

## Differential evolution algorithm

**Fig 1:**
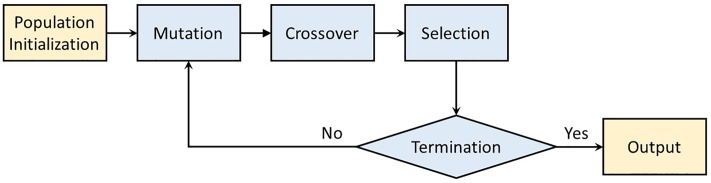
Flow chart of differential evolution algorithm.

Similar to other evolutionary algorithms, the DE algorithm has several operations, including population initialization, mutation, crossover and selection. The whole process is shown in Fig 1.

### Population initialization

The initialization operation aims to generate the first generation for the iterative process. If there is no priori knowlege about the optimum, the population can be initialized randomly. In this case, the individuals are widely distributed. Usually, the uniform distribution is used.

### Mutation operation

In many evolutionary algorithms, the mutation operation generates new vectors via adding disturbance to the individuals in the current generation. In DE, the disturbance is ralated with the difference between two other randomly selected individuals. The generated vectors are called mutant vectors. This operation enhances the search capability, and it is the key characteristic of the DE algorithm.

### Crossover operation

The crossover operation is used to increase diversity of the population. It reassigns new values to some components of the mutant vector. The components are selected randomly, and the probability is controled by a parameter, i.e. crossover rate. The result new vector is named as crossover vector.

### Selection operation

The selection operation generates population of next generation from the crossover vectors and the original individuals in the current generation. Usually, the DE algorithm utilizes greedy algorithm to select new individuals according to the fitness.

As shown in Fig 1, the mutation, crossover and selection are performed iteratively. When the termination criteria is meet, the process stops. And the algorithm outputs the best individual of the last generation.

## Point cloud registration based on differential evolution algorithm

The original TrICP algorithm estimated the rigid transformation and the overlapping rate using a iterative procedure. Such procedure could fall into a local optima easily. Therefore, it needs a good initial value. However, selection of the initial value is a difficult problem. In this paper, we turn to use a global optimization method. Due to efficiency and accuracy, the DE algorithm is increasingly popular in optimization field. Thus, it is adapted for registration partially overlapping point sets.

### Formulation

Let $M={\left\{m_{i}\right\}}_{i=1}^{N_{M}}(m_{i}\in R^{n},i=1,\cdots ,N_{M})$ be the reference point set, and $D={\left\{d_{j}\right\}}_{j=1}^{N_{D}}(d_{j}\in R^{n},j=1,\cdots ,N_{D})$ be the registration point set. The registration problem is to find the optimal rotation matrix *R* and the transform vector *t* to achieve the best alignment with two point clouds. Denot *m*_*c*(*j*)_ as the corresponding point of *d*_*j*_ in *M*. If the rotation *R* and the translation *t* between the two point sets can be obtained, the error between the two matched points can be computed as,

$$\begin{array}{c} e\left(d_{j}\right)={∥Rd_{j}+t-m_{c(j)}∥}_{2} \end{array}$$ (1)

Then, the registration process is to minimize total errors.

In the overlapping case, only a part of *D* can be aligned with a part of *M*. Thus the overlapping rate *r* need to be estimated as well. Then the overlapped part of *D* can be defined as

$$D_{r}=\left\{d_{j}∥\max e\left(d_{j}\right)<\min e\left(d_{i}\right),d_{i}\in \left(D\backslash D_{r}\right)\right\}$$

Then the TrICP algorithm find the optimal *R*, *t* and *r* by

$$\begin{array}{c} \min_{R,t,r}\frac{1}{N_{D_{r}}r^{1+\lambda }}\sum_{d_{j}\in D_{r}}{∥Rd_{j}+t-m_{c(j)}∥}_{2} \end{array}$$ (2)

where *N*_*r*_ is the number of points in *D*_*r*_, and λ is a preset parameter (in this paper, λ = 2).

**Fig 2:**
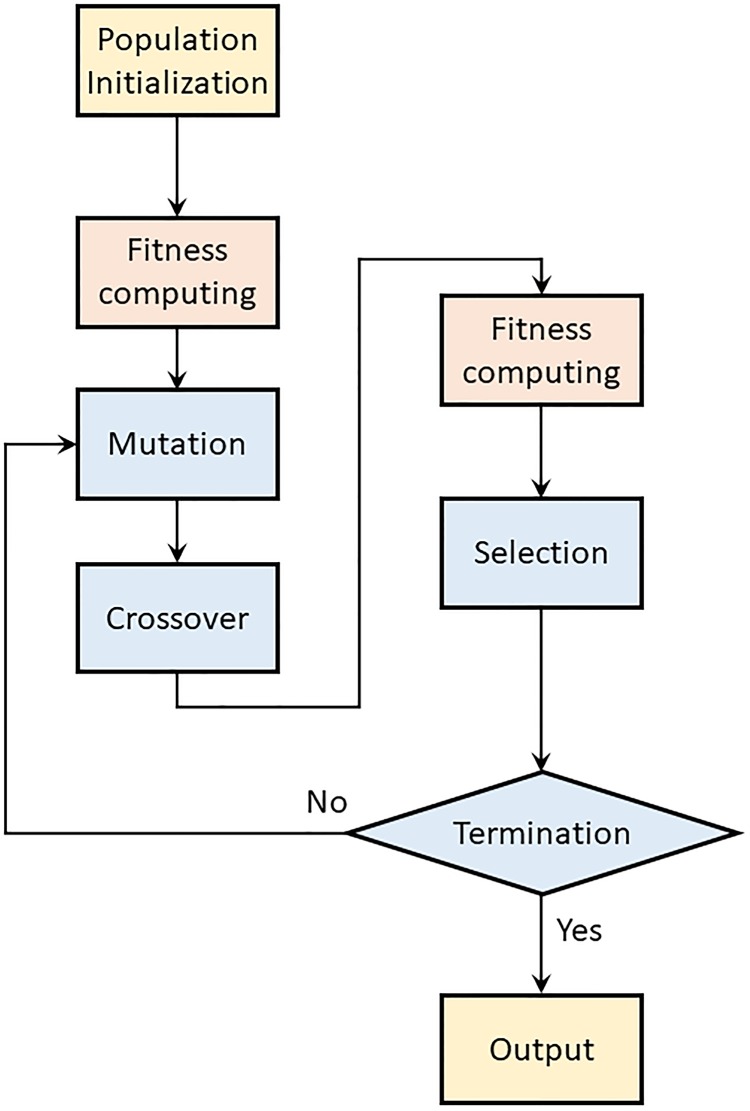
Flow chart of the proposed algorithm.

In the iterative process of DE, the objective function in Eq 2 can be seen as the fitness function which measures the goodness of the individuals. Then, the whole process of registration with DE algorithm is shown in Fig 2.

### Initialization

Let *x*_*i*_(*g*) represent the *i*^*th*^ individual in the population of the *g*^*th*^ generation. In this paper, each individual is a 7-dimensional vector, containing a 3D translation vector *t*, a 3D rotation vector and a trimming parameter. The rotation matrix *R* can be converted from the rotation vector. And the first generation can be computed using Eq (3).

$$\begin{array}{c} x_{j,i}\left(0\right)=x_{j,i}^{L}+rand\left(0,1\right)\cdot \left(x_{j,i}^{U}-x_{j,i}^{L}\right) \end{array}$$ (3)

where *x*_*j*,*i*_(0) is the *j*^*th*^ gene in the *i*^*th*^ individual, and $x_{j,i}^{U}$ and $x_{j,i}^{L}$ are the upper and lower bound of each gene. *rand*(0, 1) generates a random number which is uniformly distributed between 0 and 1.

### Calculating transformation and fitness

The operations in following steps, such as mutation and selection, are based on the fitness of individuals. Moverover, we also perform optimization like what is done in TrICP, which is utilized to improve the fitness. Thus, the following operations will be performed on a population which has higher quality.

In this step, We first transform the cloud *M* to *D* by initial transformation, and find the correspondence between the points in two sets. Secondly, we discard the point pairs if the distance between corresponding points is out of scope defined by initialized upper and lower threshold. Thirdly, the errors of the left points is calculated and the overlapping rate can be obtained. Finally, we find the index with smallest overlap rate, and update the minimum overlap rate and previous overlap rate. We then replace the two point clouds by the trimmed ones. For new point clouds, the transformation matrix can be calculated. Then the rotation matrix *R*′ and the translation vector *t*′ can be obtained by singular value decomposition (SVD).

### Mutation

In the mutation operation of original DE algorithm, the involved individuals are selected randomly. And the operation is performed as

$$\begin{array}{c} v_{i}\left(g\right)=x_{r1}\left(g\right)+F\cdot (x_{r2}\left(g\right)-x_{r3}\left(g\right)) \end{array}$$ (4)

where *r*1 ≠ *r*2 ≠ *r*3 ≠ *i* are indices of individuals which are selected randomly, *F* is the mutation scale factor, *v*_*i*_(*g*) is the mutation vector in *g*^*th*^ generation.

Obviously, the operation in the form of Eq 4 does not consider the evolutionary state of the current generation. It has same effect on all the individuals in spite of the fitness. Therefore, the best individual in this generation is introduced. Then the operation has a form as

$$\begin{array}{c} v_{i}\left(g\right)=x_{r1}\left(g\right)+F_{1}\cdot (x_{r2}\left(g\right)-x_{r3}\left(g\right))+F_{2}\cdot (x_{best}\left(g\right)-x_{r1}\left(g\right)) \end{array}$$ (5)

However, this operation has similar effect on all the individuals in spite of the fitness. Actually, the individuals which have good fitness, is more likely to trapped into a local optimum. We should add more disturbance. For the individuals, which has bad fitness, should be changed toward good ones. That is the mutation scale vectors in Eq 5 must adjusted according to the fitness of the individual. In this paper, we introduce a new operator shown in Eq 6

$$\begin{array}{c} v_{i}\left(g\right)=x_{r1}\left(g\right)+F\cdot \gamma_{1}\cdot (x_{r2}\left(g\right)-x_{r3}\left(g\right))+F\cdot \gamma_{2}\cdot (x_{best}\left(g\right)-x_{r1}\left(g\right)) \end{array}$$ (6)

Here, *F* is the current scale factor. *γ*_1_ and *γ*_2_ are the weights, which adjusted according to the fitness of the individuals. They can be computed as

$$\gamma_{1}=\frac{f-f_{best}}{f_{worst}-f_{best}}$$ (7)

$$\gamma_{2}=1-\gamma_{1}$$ (8)

where *f* is the fitness of the current selected individual, *f*_*best*_ and *f*_*worst*_ are the best fitness and worst fitness in the current generation respectively. Since the problem is minimization, *f*_*best*_ is the smallest value. Therefore, when the individual is bad, i.e. its fitness value is large, the λ_1_ will be greater than λ_2_. The mutation is more likely toward to the good individuals. Otherwise, when the individual is good, the operation is like the original one which has greater randomness.

Moreover, to keep the population from early-maturing, we change the value adaptively as following,

$$\begin{array}{c} F=F_{0}\cdot 2^{\lambda } \end{array}$$ (9)

and,

$$\begin{array}{c} \lambda =e^{1-\frac{G}{G+1-g}} \end{array}$$ (10)

*F*_0_ is the basic mutation scale factor, *G* is the number of generations, and *g* is the index of current generation.

### Crossover

In this paper, the uniform crossover is used. For the individuals in the *g*^*th*^ generation, the crossover operation is performed as follows.

$$\begin{array}{c} u_{j,i}\left(g+1\right)=\{\begin{array}{cc} v_{j,i}\left(g+1\right) & \text{if}\;rand(0,1)<CR\;\text{or}\;j=j_{rand}\text{,}\\ x_{j,i}\left(g\right) & \text{otherwise} \end{array} \end{array}$$ (11)

where *CR* ∈ (0, 1) is the corssover rate. It controls the proportion of the components where the crossover occurs. *j*_*rand*_ is the index of randomly selected component. This insures that crossover occurs on at least one of the components. The generated vector is called crossover vector.

### Selection

The DE algorithm applies greedy strategy to select individuals which have good fitness for the next generation.

$$\begin{array}{c} x_{i}\left(g+1\right)=\{\begin{array}{cc} u_{i}\left(g+1\right) & \text{if}\;f(u_{i}\left(g+1\right))\leq f(x_{i}\left(g\right))\text{,}\\ x_{i}\left(g\right) & \text{otherwise} \end{array} \end{array}$$ (12)

where *f*(⋅) is the fitness of the individual. The fitness can be obtained by TrICP (Eq 2). This operation generates population for the *g* + 1 generation.

## Experiment

In this section, the proposed algorithm was verified on a public and popular 3D range data set, i.e. the Stanford 3D Scanning Repositor [19]. And the proposed algorithm was compared with three related methods: the registration based Particle Filters (PF) [16], the registration based on GA [20], and the registration based on original DE. And we denote these approach as PF, GA, and DE respectively.

### Convergence speed

For those global optimization based algorithms, the convergence speed is an important indicator of the algorithm’s performance. Therefore, we selected two shapes from S1 Dataset for the experiment. One was the bunny shape and the other was the dragon shape. To generate data set, a part of the points in the shape were discarded. And after adding white noises to the position of points, the left points formed the data set. Then another part of the shape was cut down. And a randomly generated transformation (*R*_*r*_, *t*_*r*_) was applied to the left part. The result point set was the reference set. Then we used the four methods to register the data set and model set.

**Fig 3:**
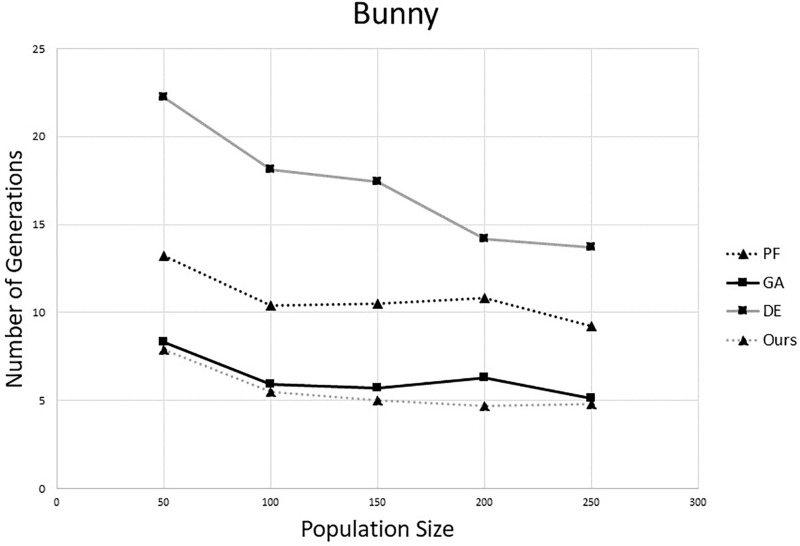
Comparison of number of generations on “Bunny”. (The overlapping rate is 75%.)

**Fig 4:**
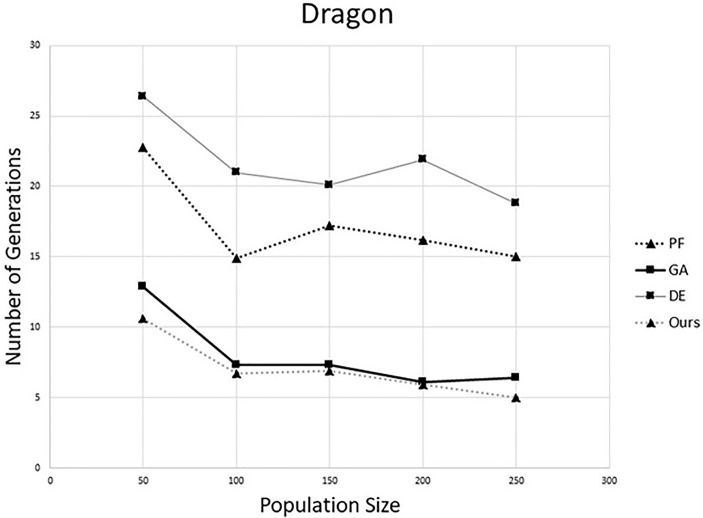
Comparison of number of generations on “Dragon”. (The overlapping rate is 75%.)

To compare the performance on convergence speed, we fixed up the overlapping rate (*r* = 0.75) and varied the population size in different algorithms. We generated 10 pairs sets using the process mentioned in the last paragraph. Then, each algorithm was tested on all pairs. The average number of iterations of each algorithm is shown in Figs 3 and 4. From these results, one can notice that the proposed method need fewest generations than other three methods. This is because in the proposed method, the individuals were evolved according to their own state. So the search ability of the population is better than others. Also, comparing with the original DE, the proposed method can increase the efficiency greatly.

### Overlapping rate

Then we fixed up the population size for all the methods, and varied the overlapping rate between the data set and the reference set. In this experiment, the population size was set to 100 and the bunny shape from S1 Dataset is used. All the methods were tested with 5 different overlapping rates. And the tests were performed for 30 trials. In this case, the successful rate and the number of iterations are concerned. The result is shown in Table 1. As shown by the results, the propose approach is more robust than other three methods.

**Table 1:** Performance comparison under varying overlapping rate. Suc is the percentage of successful trials, and NG is the average number of generations.

Method	*r* = 1		*r* = 0.85		*r* = 0.7		*r* = 0.6		*r* = 0.5	
	Suc	NG	Suc	NG	Suc	NG	Suc	NG	Suc	NG
PF	100	10.3	97	12.73	93	14.53	80	19.47	50	23.63
GA	100	5.13	100	6.17	93	10.37	80	15.93	50	18.73
DE	100	13.5	97	16.53	67	19.7	20	24.53	10	28.9
Ours	100	4.17	100	4.5	97	8.1	87	10.77	63	14.23

**Fig 5:**
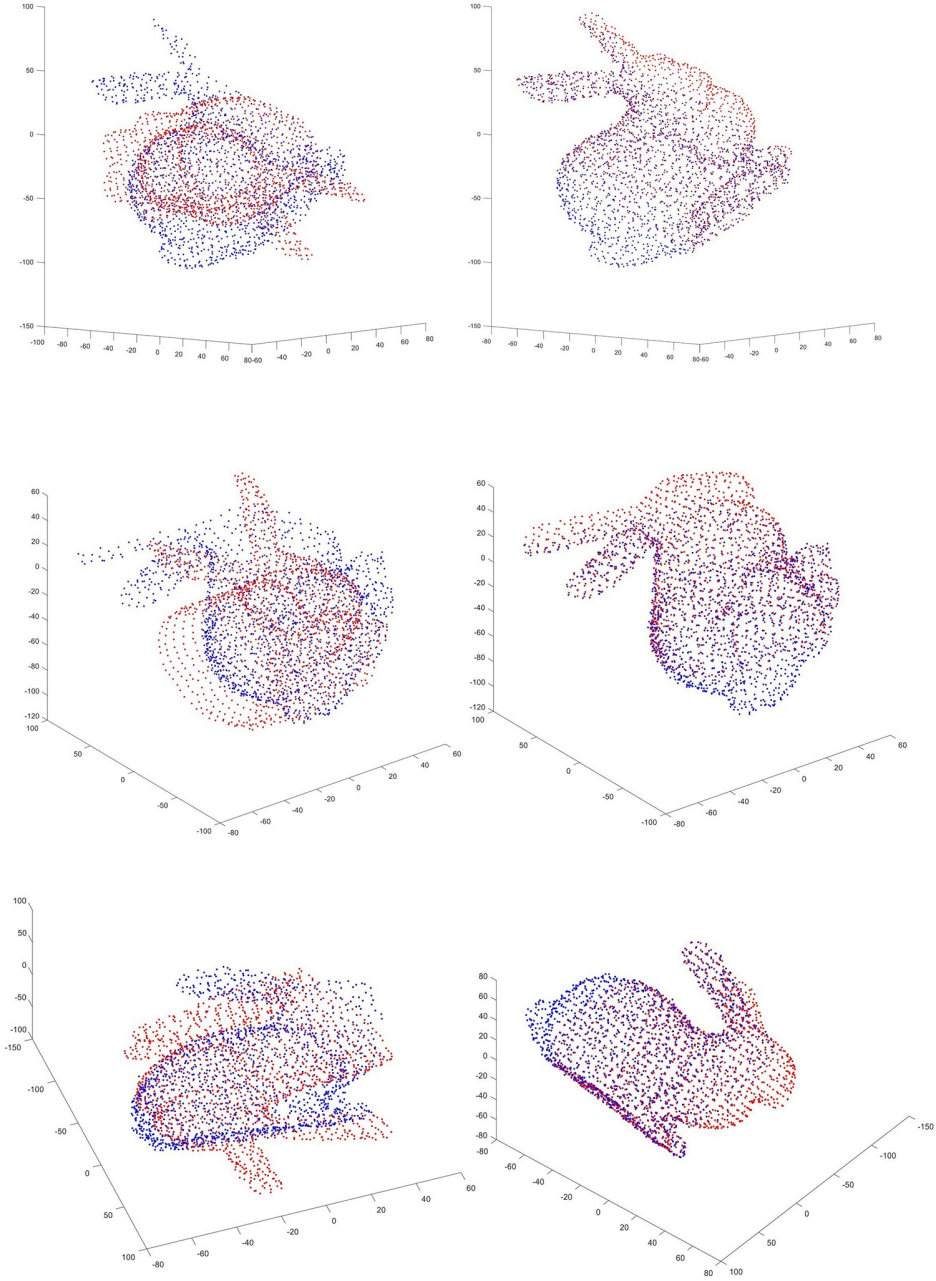
Registration results of the proposed algorithm for shape “Bunny”. The first column is the initial position of two point sets. And the second column is the results after registration using the proposed algorithm.

**Fig 6:**
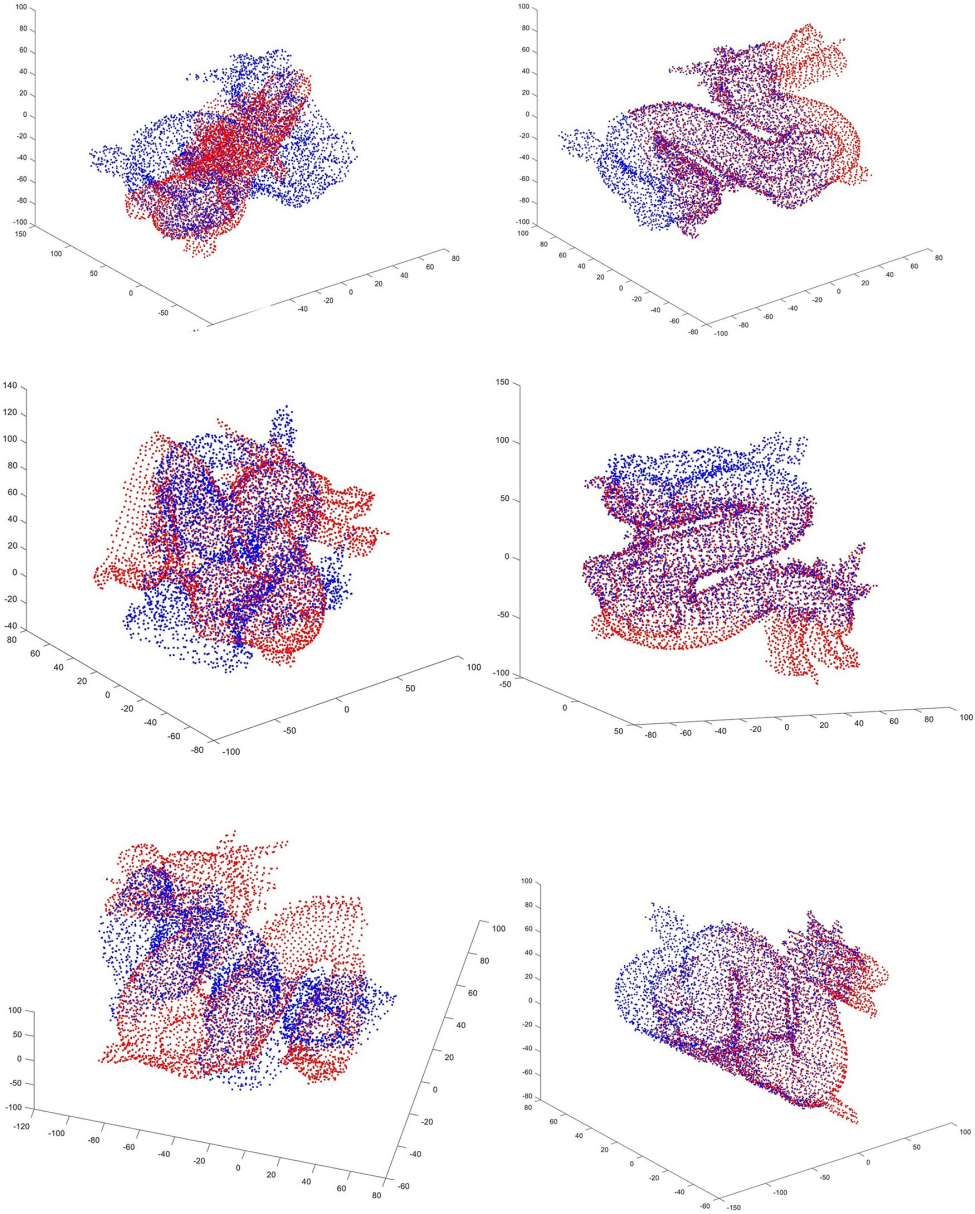
Registration results of the proposed algorithm for shape “Dragon”. The first column is the initial position of two point sets. And the second column is the results after registration using the proposed algorithm.

In summary, the proposed approach can find optimal result in fewer generations and is more robust for the registration between partially overlapped point cloud. Figs 5 and 6 gives some examples of registration results.

## Conclusion

In this paper, we present a new variant of TrICP algorithm based on the differential evolution algorithm. In the proposed algorithm, the DE algorithm was used to obtain a global optimal solution. Via designing appropriate evolutionary operations, the population can be distributed widely. Therefore, it is difficult for the proposed algorithm to be trapped into local optima. The result of the experiment showed that the proposed algorithm can obtain accurate transformation between two point clouds, even when the overlapping rate is low. Meanwhile, the computational cost is relative low comparing with common algorithms such as genetic algorithm and particle filtering.

## Supporting information

S1 Dataset The minimal data set. (ZIP)
